# Calomel and Potassium Bromide

**Published:** 1895-07-06

**Authors:** 


### CALOMEL AND POTASSIUM BROMIDE.

L. N. Thompson deals with the following prescription (Pharm. Journ., February 24th, 1894): ℞ potass. bromid, gr. x.; calomel, gr. iii.; m. ft. pulv. mitte tales xii. As soon as the ingredients are rubbed together the mixture begins to darken, and if water be present becomes instantly greyish-black. This is a dangerous incompatibility, as a new mercuric salt is formed which is a powerful poison. The dark colour is due to the simultaneous separation of metallic mercury. Decomposition occurs as follows: $2\ Hg\ Cl + 4\ K\ Br = Hg\ Br_2 + 2\ K\ Br + Hg + 2K\ Cl$. There is no method by which the decomposition can be avoided, and calomel and potassium bromide should, therefore, never be prescribed together.

